# Monoacylglycerides from the Diatom *Skeletonema marinoi* Induce Selective Cell Death in Cancer Cells

**DOI:** 10.3390/md17110625

**Published:** 2019-11-01

**Authors:** Marco Miceli, Adele Cutignano, Mariarosaria Conte, Raffaella Ummarino, Alessandra Romanelli, Menotti Ruvo, Marilisa Leone, Flavia Anna Mercurio, Nunzianna Doti, Emiliano Manzo, Giovanna Romano, Lucia Altucci, Adrianna Ianora

**Affiliations:** 1 CEINGE-Biotecnologie Avanzate s.c.ar.l., 80145 Naples, Italy; micelim@ceinge.unina.it; 2 CNR-Institute of Biomolecular Chemistry, Via Campi Flegrei 34, Pozzuoli, 80078 Naples, Italyemanzo@icb.cnr.it (E.M.); 3 Department of Precision Medicine, University of Campania ‘Luigi Vanvitelli’, Via L. De Crecchio 7, 80138 Naples, Italy; mariarosaria.conte@unicampania.it; 4 Institute of Biostructures and Bioimaging (IBB-CNR), Via Mezzocannone 16, 80134 Naples, Italy; raffaella.u@libero.it (R.U.); menotti.ruvo@unina.it (M.R.); marilisa.leone@cnr.it (M.L.); flaviaanna.mercurio@unina.it (F.A.M.);; 5 Department of Pharmaceutical Sciences, University of Milan, via Venezian 21, 20133 Milan, Italy; 6 Marine Biotechnology Department, Stazione Zoologica Anton Dohrn, Villa Comunale, 80121 Naples, Italy; adrianna.ianora@szn.it

**Keywords:** diatoms, *Skeletonema marinoi*, cytotoxic activity, NMR, MS, bioactive lipids

## Abstract

Microalgae are an excellent source of valuable compounds for nutraceutical and cosmeceutical applications. These photosynthesizing microorganisms are amenable for large-scale production, thus overcoming the bottleneck of biomass supply for chemical and activity characterization of bioactive compounds. This characteristic has recently also prompted the screening of microalgae for potential pharmaceutical applications. Here, we show that monoacylglycerides (MAGs) purified from the marine diatom *Skeletonema marinoi* have selective cytotoxic activity against the haematological cancer cell line U-937 and colon cancer cell line HCT-116 compared to normal MePR-2B cells. LC-MS analysis of the raw extract revealed that in their natural form, MAGs occur as 2-monoacyl derivatives and include mainly C16 and C20 analogues, but they are converted into the corresponding 1-isomers during purification processes. Pure compounds along with the synthetic 1-monoarachidonoylglycerol tested on HCT-116 and U-937 tumor cell lines induced cell death via apoptosis. The mechanism of action was investigated, and we show that it involves the induction of apoptosis through caspase 3/7 activation. These findings pave the way for the possible use of these molecules as potential anticancer agents or as precursors for the generation of new and more potent and selective compounds against tumor cells.

## 1. Introduction

Microalgae are a group of heterogeneous and diverse microorganisms representing an excellent source of pigments, lipids, carotenoids, ω-3 fatty acids, polysaccharides, vitamins, and other fine chemicals for nutraceutical applications [1]. Recently, with the growing demand of additional compounds for use in medicine and increased incidence of cancer and other human pathologies, compound mixtures extracted from microalgae have also been screened for active ingredients with potential pharmaceutical applications. The interest in microalgae also arises from the possibility of cultivating them in large quantities, thus overcoming the problem of supply for chemical and bioactivity characterization, and, if required, industrial production. However, although a range of interesting properties have been observed in microalgal extracts that could be of potential interest for the development of new anti-inflammatory, anticancer, and antibiotic drugs [2,3,4,5,6,7,8,9,10], active principles often remain unknown [2,4,6,8,9]. For example, organic extracts of the green alga *Chlorella ovalis* and the dinoflagellate *Amphidinium carterae* suppressed the growth of HL-60 cells (human promyelocytic leukemia cell line) [2]. Fractions from a methanolic extract of the green algae *Nannochloropsis oculata* had cytotoxic activity on HL-60, A-549, HEP-3B, HCT-116, and SW-480 cancer cells [9], and extracts from the diatom *Chaetoceros calcitrans* had cytotoxic effects on human breast cancer cell lines (MCF-7) [4]. Extracts of *Skeletonema marinoi* have shown bioactivity against human melanoma A2058 cells when cultured at low temperature-high light [11] or under nitrogen-starvation conditions [8].

In other studies, pure molecules, such as two monogalactosyl diacylglycerols, isolated from the diatom *Phaeodactylum tricornutum*, showed potential anticancer effects by specifically inducing apoptosis in a human cancer cell line [12]. Also, the fatty alcohol ester nonyl 8-acetoxy-6-methyloctanoate (NAMO) from the same diatom species showed bioactivity on three different cancer cell lines, including human leukemia (HL-60) lung carcinoma (A-549), and a mouse melanoma (B16F10) [2,13]. Miralto et al. [14] reported that diatom unsaturated aldehydes had antiproliferative effects on Caco-2 cells derived from a human colon adenocarcinoma; Sansone et al. [15] showed similar effects on colon COLO-205 and lung A-549 adenocarcinoma cell lines. Manzo et al. [16,17] claimed immunomodulatory activity of Sulfavant A, a synthetic glycolipid, which is a prototype for a new class of molecular vaccine adjuvants based on a sulfolipid skeleton and structurally inspired by natural α-D-sulfoquinovosyl-diacylglycerols purified from the marine diatom *Thalassiosira weissflogii*. Furthermore, from the same diatom species, very recently, a novel phosphatidylmonogalactosylglycerol lipid (PGDG) rich in polyunsaturated fatty acids (PUFA) was isolated, which showed immunostimulatory activity mediated by Toll-like receptor-4 in dendritic cells [18].

Here, we report on the potential anticancer activity and chemical profiling of bioactive extracts and fractions purified from the diatom *Skeletonema marinoi,* which showed selective cytotoxic activity against the hematological cancer cell line U-937 and the colon cancer cell line HCT-116 compared to normal human mesenchymal progenitor model MePR-2B cells. This species was identified as the most active following the screening of several microalgal extracts in the frame of a National Operational Program for Research and Competitiveness (http://www.ponrec.it/open-data/risultati/ricerca-industriale/PON01_02782) to identify molecules with antiproliferative activity against circulating tumor cells. In the present study, we show that the active fraction we obtained from *S. marinoi* contains a pool of monoacylglycerides mainly with C16 and C20 alkyl chains. Isolated molecules, commercially available monoacylglycerides identified in the fraction, and a synthetic analogue, i.e., 1-monoarachidonoylglycerol, were evaluated individually for their bioactivity and for their ability to induce apoptosis.

## 2. Results

### 2.1. Screening of Microalgal Extracts for Potential Anticancer Activity

**Figure 1:**
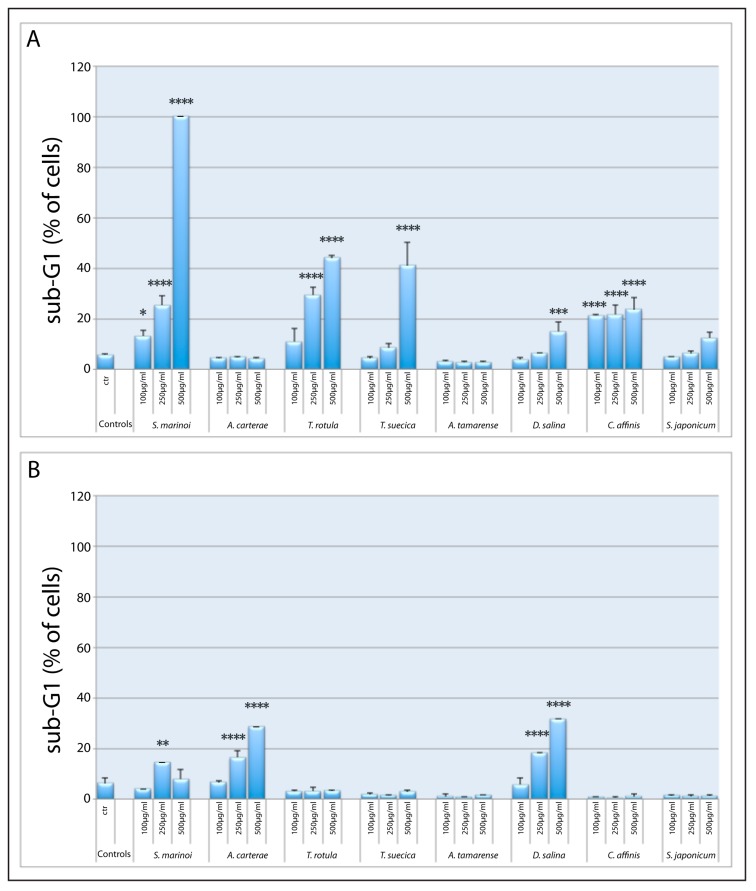
Percentage of cells in sub-G1 phase after 48-h treatment with microalgae extracts in U-937 cell line (**A**) and in MePR-2B (**B**) cell lines. All bars represent means ± SD with *n* = 3. Statistical notations: * *p* < 0.05; ** *p* < 0.01; *** *p* < 0.001; **** *p* < 0.0001. (ANOVA, with Tukey’s post-test).

Methanol extracts of microalgal species from the culture collection of the Stazione Zoologica Anton Dohrn were tested for their cytotoxic effects against a leukemia cancer (U-937) cell line in comparison to a normal cell line (MePR-2B) after 48 h (Figure 1A,B). Table S1 shows the list of species selected from the culture collection of the Stazione Zoologica with the relative identification code. Most species, except for the dinoflagellates *Amphidinium carterae* and *Alexandrium tamarense* and the diatom *Skeletonema japonicum*, showed a dose-dependent cytotoxic activity against U-937 cells. Of these, the most active were the diatoms *Skeletonema marinoi* and *Thalassiosira rotula*, and the green alga *Tetraselmis suecica* (Figure 1A).

**Figure 2:**
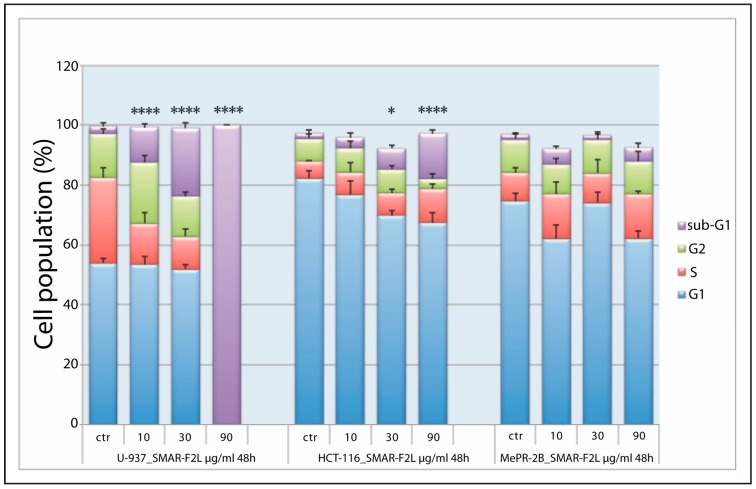
Cell cycle distribution. Percentage of cell population in different phases of the cell cycle for U-937 (group on the left), HCT-116 (group in the middle), and MePR-2B cells (group on the right) after treatment for 48 h with fraction SMAR-F2L at 10, 30, and 90 µg/mL concentrations. All bars represent means ± SD with *n* = 3. Statistical notations for differences in cell populations in sub-G1 compared to control: * *p* < 0.05; ** *p* < 0.01; *** *p* < 0.001; **** *p* < 0.0001. (ANOVA, with Tukey’s post-test).

In particular, *S. marinoi* extract showed the highest activity, inducing 100% blockage of cells in the sub-G1 phase of the cell cycle after 48 h at extract concentrations of 500 µg/mL, without affecting normal MePR-2B cells at any of the concentrations tested. The presence of a peak corresponding to the sub-G1 phase, with no appreciable presence of cells in G1, S, and G2 cell cycle phases (Figure 2), suggests that the main action was induction of cell death. 

Conversely, *A. carterae* and the green alga *Dunaliella salina* showed a perceptible sub-G1 peak, indicating cell death at the highest concentrations in normal cells (Figure 1B) compared to the U-937 cell line. Based on these results, we selected the diatom species *S. marinoi* for further studies.

### 2.2. Fractionation of Skeletonema marinoi Diatom Extract by Silica Gel Chromatography and HPLC Purification of MAGs 

The raw methanol extract from *S. marinoi* was fractionated by silica gel column chromatography with a petroleum ether/diethyl ether gradient and collected fractions were tested for cytotoxic activity on U-937 and HCT-116 cells in comparison with normal cell MePR-2B. Figure 2 shows the effect of fraction SMAR-F2L at different concentrations on cell cycle phase distribution in the U-937, HCT-116, and MeBR-2B cell lines after 48 h. In Supplementary Figure S1, cell cycle distribution in the three different cell lines after 24 and 72 h is also shown.

**Figure 3:**
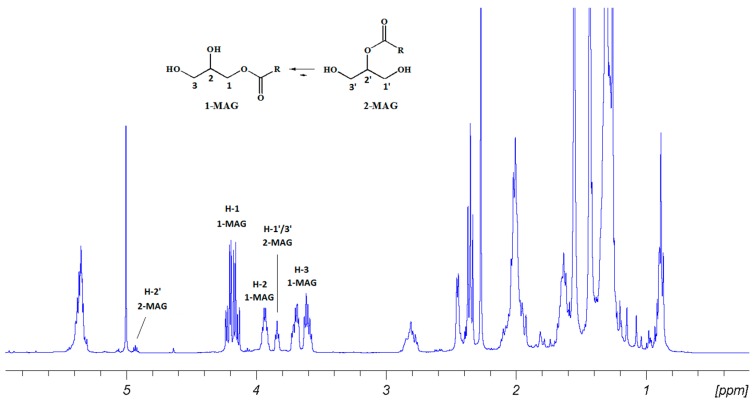
^1^H-NMR (400 MHz, CDCl_3_) spectrum of the bioactive fraction (SMAR-F2L) from the diatom *Skeletonema marinoi*.

**Figure 4:**
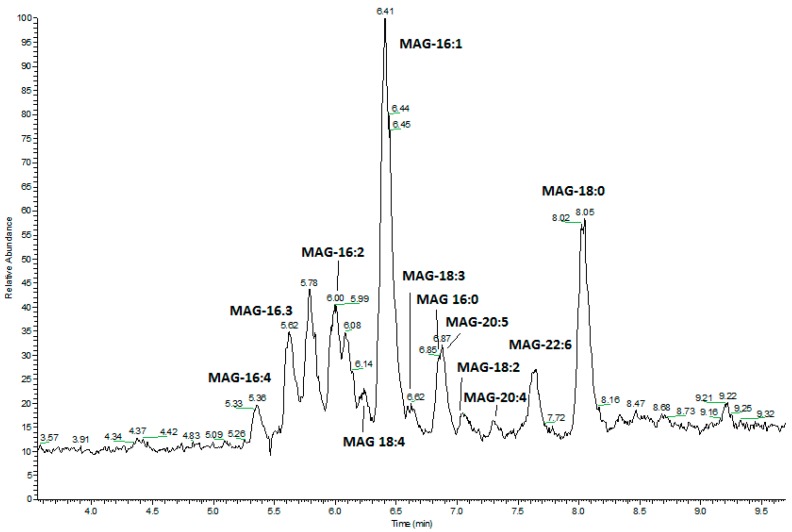
UHPLC-ESIMS profile of the bioactive fraction (SMAR-F2L) from the diatom *Skeletonema marinoi*.

U-937 cells treated with pooled fractions eluted with diethyl ether (EE) 100% (SMAR-F2L) were already totally blocked in the sub-G1 phase at 24 h at 90 µg/mL (Supplementary Figure S1A), reaching 100% after 48 h at this concentration (Figure 2). After 72 h, the effect was more pronounced, since cells treated with 30 µg/mL were all dead (Supplementary Figure S1B). HCT-116 cells showed a significant proportion of cells in the sub-G1 phase (*p* < 0.0001) at the same concentration, but only at 72 h (Supplementary Figure S1B) since these cells are more resistant to treatments. The active fraction had no effect on MePR-2B normal cells, underlying the specific cytotoxic effect on tumor cells. NMR data recorded for this fraction revealed diagnostic signals of monoacylglycerides (MAGs) occurring as a mixture of 1-/2-isomers at a ratio of 9:1 (Figure 3 and Supplementary Figures S2–S5). In particular, a COSY experiment, together with signals belonging to fatty acid chains, delineated two spin systems attributed to monosubstituted glycerols. The main set of signals was attributed to 1-MAGs and was constituted by deshielded acylated oxymethylene protons at δ 4.21 (dd, 11.7, 4.7 Hz) and 4.15 (dd, 11.7, 6.2 Hz) (H_2_-1) coupled with the oxymethine at δ 3.93 (m, H-2), in turn correlating with the two methylene protons at δ 3.62 (dd, 11.3, 5.4 Hz) and 3.42 (dd, 11.3, 4.3 Hz) (H_2_-3) (Figure 3, Figure S2). The second set was assigned to a 2-substituted glycerol unit and consisted of an acylated oxymethine at δ 4.93 (m, H-2’) coupled with the four magnetically equivalent methylene protons at positions 1’ and 3’ of the glycerol at δ 3.84 (m, H_2_-1’/H_2_-3’). The LC-MS profile suggested that the MAG mixture was composed of unsaturated C16, C20:5 (eicosapentaenoic acid, EPA), and C22:6 (docosahexaenoic acid, DHA) fatty acids, with a minor occurrence of monoacylglycerols containing unsaturated C18 and C20:4 (arachidonic acid, ARA) fatty acids (Figure 4). On the whole, this fatty acid (FA) composition mirrored that profiled by GC-MS after methanolysis of complex lipids (Supplementary Table S2).

**Figure 5:**
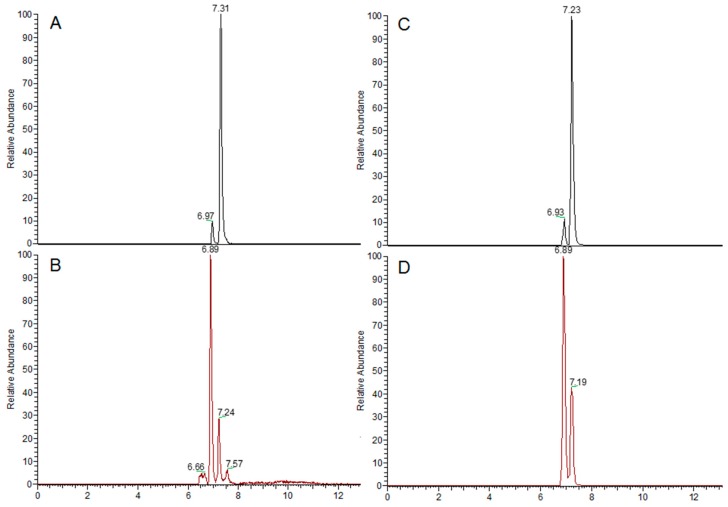
Extracted ion chromatograms (XICs) at *m/z* 401.26623 corresponding to MAG-ARA in: (**A**) purified fraction of the diatom *Skeletonema marinoi*; (**B**) methanol extract of *S. marinoi*; (**C**) a synthetic standard of 1-MAG-ARA; (**D**) XIC at *m/z* 406.29861 of a commercial standard of 2-MAG-ARA*_d5_*.

As an example, Figure 5 shows a comparison of extracted ion chromatograms (XIC) for M + Na^+^ ion at *m/z* 401.26623 corresponding to monoarachidonoylglycerol in both the silica fraction and methanol extract (Figure 5A and B, respectively) together with those of a synthetic standard of 1-monoarachidonoylglycerol (MAG-ARA-Figure 5C) and a commercial standard of 2-monoarachidonoyl-*d*_5_ glycerol (MAG-ARA*_d5_*, M + Na^+^ *m/z* 406.29861, Figure 5D). The difference in the retention time of the two isomers is clearly detectable.

In order to obtain pure MAGs for cytotoxicity studies, we carried out a HPLC purification of the bioactive fraction. For this purpose, a series of peaks corresponding to natural MAGs were collected, including C16:4, C16:3, C16:2, C16:1, and a mixture of EPA/C18:3 derivatives (Supplementary Figure S6–S9). Due to its inherent chemical instability, MAG-C16:4ω1 degraded during sample manipulation. In addition, MAG-DHA was not isolated in a sufficient amount for further processing. Since the monoacylglyceride of EPA was isolated together with C18:3 derivative, we used the synthetic derivative 1-monoarachidonoyl glycerol (1-MAG-ARA) as a representative of polyunsaturated MAG as described below. Due to their commercial availability, 1-palmitoleoyl-*rac*-glycerol (MAG-C16:1), 1-Palmitoyl-*rac*-glycerol (MAG-C16:0), and 1-stearoyl-*rac*-glycerol (MAG-C18:0) were purchased as pure standards for the cytotoxicity tests.

### 2.3. Biological Activity of 1-MAGs 

**Figure 6:**
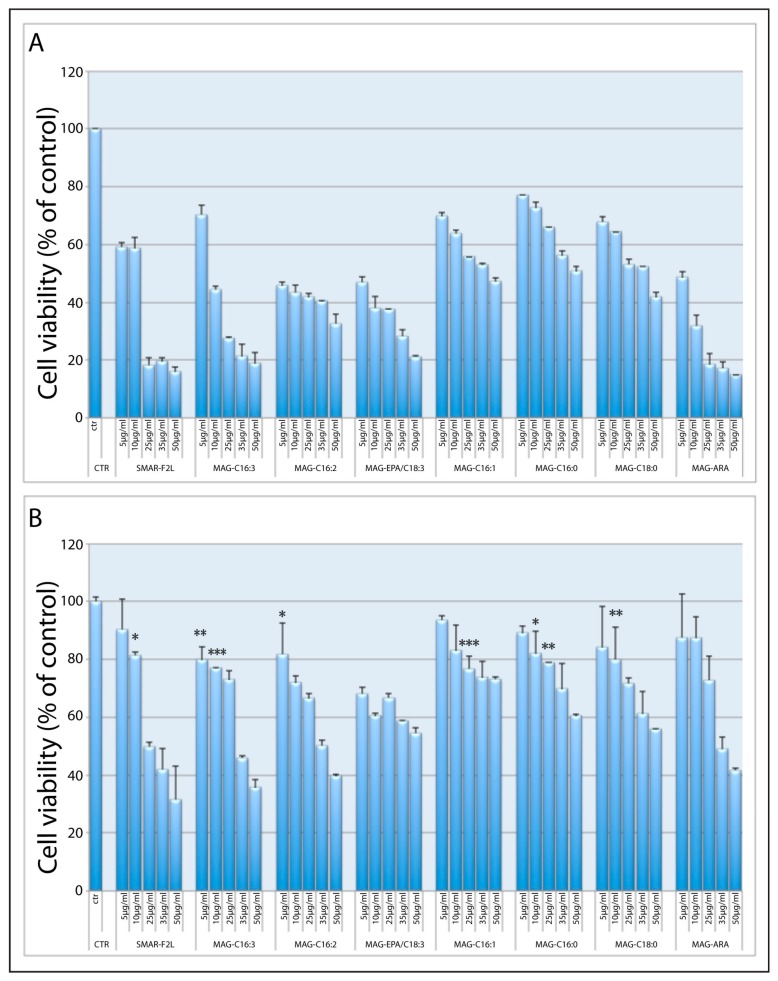
Cell viability assessed by the MTT assay, expressed as % of the control. U937 (**A**) and HCT116 (**B**) cells after 48 h of treatment with different MAGs at 5, 10, 25, 35, and 50 µg/mL. The dose-dependent decrease in cell viability can be seen as a decrease in bar height. All bars represent means ± SD with *n* = 3. Statistical notations: * *p* < 0.05; ** *p* < 0.01; *** *p* < 0.001. Note that in panel (**A**) all data were highly significant (*p* < 0.0001), but are not indicated to avoid overcrowding of symbols. Similarly, in panel (**B**) extremely significant differences (*p* < 0.0001), which are the majority, are not indicated to avoid overcrowding of symbols (ANOVA, with Tukey’s post-test).

Purified and commercial MAGs were tested using U-937 and HCT-116 cancer cell lines and the normal MePR-2B cells. Cell viability assays were performed after 24 and 48 h of treatment at increasing concentrations of 5, 10, 25, 35, and 50 µg/mL (Figure 6 and Supplementary Figure S10). As shown in Figure 6A and in Supplementary Figure S10, the hematological U-937 cell line is strongly susceptible to treatment in a time- and concentration-dependent manner. Indeed, a considerable reduction of leukemic cell viability can already be detected at the lowest concentration of 5 µg/mL, both after 24 (Figure S10A, *p* < 0.0001) and 48 h (Figure 6A, *p* < 0.0001) of treatment. The effect on the colon cancer cell line HCT-116, which is a very resistant form of tumor, was weaker, although a significant decrease in cell viability was detectable at a concentration of 10 µg/mL at 48 h for most of the compounds, with MAG-C16:3 (*p* < 0.001), MAG-C16:2, and MAG-EPA/C18:3 (*p* < 0.0001) being the most active at this concentration (Figure 6B). In both cancer cell lines, purified MAG-C16:3 showed a slightly higher activity compared to the others. On the other hand, we did not detect relevant effects on MePR-2B cells at 24 h (Supplementary Figure S11A), underlying the cancer-specific activity of these derivatives. After 48 h, MAG-C16:3 and MAG-C16:2 at the highest concentrations slightly affected the cell viability of MePR cells (Supplementary Figure S11B).

Results were corroborated by trypan blue exclusion assay to assess cell proliferation (Supplementary Figure S12 and Supplementary Table S3). All MAGs significantly affected (*p* < 0.0001) U-937 cell proliferation already after 24 h of incubation at 25 µg/mL. The proliferation of HCT116 was also significantly affected after 24 h of incubation (*p* < 0.0001), with MAG-C16:0 showing a slightly weaker activity (*p* < 0.001). MePR-2B proliferation was not affected at 24 h while it was slightly reduced after 48 h of treatment. The effect was weaker compared to the effect of MAGs on the two tumor cell lines, though the difference with respect to the control was significant (Supplementary Figure S12).

**Figure 7:**
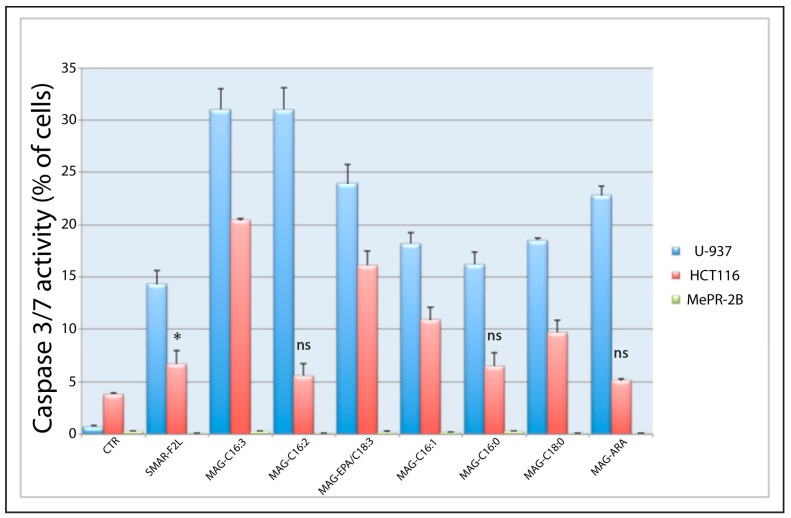
Caspase 3/7 activation in U937, HCT-116, and MePR-2B cells after 24 h of treatment with purified MAGs and controls used at a concentration of 25 µg/mL. All bars are means ± SD with *n* = 3. Statistical notations: ns = non-significant; * *p* < 0.05. Note that only differences non-significant with respect to the control (ns) and difference with *p*-value < 0.05 (*) are indicated. All other values are significantly different (*p* < 0.0001), with respect to the control and are not indicated to avoid overcrowding of symbols (ANOVA, with Tukey’s post-test).

Furthermore, we investigated caspase 3/7 activation by purified and commercial MAGs (Figure 7) in both cancer cell lines following 24 h of treatment at 25 µg/mL. Data confirmed a specific apoptosis-mediated cell death induced in tumor cells by these derivatives produced by the diatom *S. marinoi* while no pro-apoptotic effect was observed in the normal MePR2B cell line. 

## 3. Discussion

Although targeted therapies have greatly enriched the choice of treatments against cancer, the advantages have remained limited and there is still a need for new treatments for different solid and hematological tumors.

Marine-derived compounds have revealed remarkable activity and singular modes of action, often differing from each other in structure, molecular targets, and toxicity profiles [19,20]. This diversity in biological activities and molecular mechanisms of action are of key interest since they may not only give rise to novel molecules but also to the possibility of modulating their production with different culturing methods. Among marine organisms, microalgae have recently received great attention, and extracts from these microorganisms have displayed anticancer/antiproliferative activities in different settings [10]. Nevertheless, despite an increasing effort to investigate the bioactive potential of microalgae, few studies have identified the molecules responsible for anti-tumor activity. In a recent study, a screening of 21 diatoms, 7 dinoflagellates, and 4 flagellates, grown in different culturing conditions, further underlined the great potential of diatoms as sources for bioactive molecules [8]. In this work, the authors showed that the extract from only one (FE60) of two different clones of the species *S. marinoi* was cytotoxic against human melanoma A2058 cells cultured in nitrogen-starvation conditions while the other clone (FE6) was not active in any growth condition. These findings emphasize the differences in secondary metabolism among clones of the same species and the influence of the culture condition on the production of bioactive molecules.

Our results indicate that total extract from another clone of the marine diatom *S. marinoi* (FE7)*,* grown in standard conditions, displays dose- and time-dependent induction of cell death in the hematological cancer cell line U-937. Interestingly, this action assumes features of cancer selectivity since, when tested on normal human mesenchymal cells (MePR-2B), no toxicity was revealed. By contrast, extracts from *A. carterae* and *D. salina* displayed slight activity in the normal cells MePR-2B, suggesting that potential anticancer activity in these extracts is hampered by a lack of specificity. 

Fractionation of the *S. marinoi* extract led to the identification of an active fraction that retained specificity against tumor cells, also showing activity against the colon cancer cell line HCT-116. 

NMR and MS analyses of the active fraction (SMAR-F2L) indicated the presence of 1-monoacylglycerols. Monoacylglycerols are a group of bioactive lipids commonly found in bacteria, fungi, plants, and animal tissues, occurring as 2-MAGs. Acyl migration in monoacylglycerols from position 2 to position 1 of the glycerol is a spontaneous, well-studied event that in fact hampers the isolation of pure 2-MAGs from natural sources, particularly when multiple chromatographic steps are required [21]. Therefore, after the identification and isolation of a series of 1-MAGs from the extract of *S. marinoi*, we suspected that in the natural form, these lipids occurred as 2-isomers. To explore this point, we performed a comparison of extracted ion chromatograms for each MAG species as detected by liquid chromatography-mass spectrometry in the bioactive fraction and raw extract. The latter was subjected to little manipulation, maintained at low temperatures, and analyzed soon after its preparation. Indeed, the comparison of the two LC-MS traces revealed that, as expected, in the natural form MAGs occur as 2-isomers; isolation through silica chromatography caused isomerization of the natural lipid pool of 2-MAGs with acyl migration from position 2 to 1 of the glycerol backbone, affording a thermodynamically more stable chemical form. 

The pure natural molecules obtained through HPLC fractionation, together with commercial standards and a synthetic analogue (MAR-ARA), were tested for potential anticancer activity, showing an appreciable decrease in cell viability in the leukemic cancer cell line U-937, and a lower effect in the more resistant colon cancer model HCT-116, while viability of the normal mesenchymal cell line MePR-2B was not compromised. Since normal cells have a lower proliferation rate compared to cancer cells, we hypothesize that the reduced effect of the compounds on healthy cells might be linked to their lower ability to incorporate MAGs as a consequence of their reduced metabolic activity. 

For this study, we used two cancer cell lines that are excellent model systems widely used for the screening of molecules with anticancer potentialities, both of which are resistant to cytotoxic drugs. The hematological cancer cell line U-937 showed a more pronounced response to treatment with MAGs compared to HCT-116. This difference may possibly be due to intrinsic molecular/genetic characteristics of the two cell lines, although further studies focusing on molecular pathways activated in the two cell lines are needed, in order to better understand the mechanism of action of MAGs used in this study.

Activation of caspase 3/7 only in the two cancer cell lines provides evidence that the induction of cell death is due to activation of apoptosis. It is interesting to note that unsaturated C16 MAGs are slightly more active than saturated MAGs in inducing cell death in cancer cells. Their activity is also higher than the C18 MAGs tested in this work, and comparable to MAG-ARA. Cancer selectivity following treatment with MAGs was underlined by the almost total absence of death induction in the normal mesenchymal cell line MePR-2B. 

In recent studies, MAG-ω3, such as MAG-EPA and MAG-DHA, have shown anti-inflammatory and anti-proliferative effects [22]. For example, studies on the anti-inflammatory activity of these compounds revealed a suppressive effect on bronchial hyper-responsiveness following per os administration of MAG-DHA and MAG-EPA, using *in vivo* models of allergic asthma. In addition, MAG-EPA induced apoptosis in HCT-116 cells and reduced tumor growth in a xenograft mouse model that received *per os* administration of MAG-EPA [23]. The involvement of marine omega-3 oils in the reduction of cancer risk and progression has been well established. Nevertheless, a better absorption capacity of ω-3 PUFA MAG compared to the absorption rate of the corresponding free PUFA [24] suggests that MAGs could be of greater medicinal interest for the treatment and prevention of inflammatory processes and tumorigenesis with respect to ω-3 oils [22,23]. 

In the present work, we also showed that MAG-ω6, such as MAG-ARA, and MAGs constituted by unsaturated C16 fatty acids, have anti-proliferative effects on tumor cell lines, suggesting the possible exploitation of these compounds for cancer treatment. Further studies may confirm whether monoacylglycerol-derived molecules with improved activities can be obtained from other diatom species or by chemical synthesis. Indeed, using a broader range of molecules and extending the study to other cancer cell lines may contribute to clarifying the molecular mechanisms underlying cancer selectivity. In addition, assessing the activation of specific markers for intrinsic and extrinsic apoptosis may help to further elucidate the mechanisms activated in response to MAGs in cancer cells. It is tempting to also perform *in vivo* studies in comparison with MAG-DHA and MAG-EPA using already established model systems (e.g., in [22,23]), which could provide insight on the possible use of these molecules in the prevention and cure of pathologies involving inflammatory processes, including tumorigenesis.

Since diatoms are considered a rich and renewable source for the sustainable production of bioactive compounds, it would be interesting to verify whether, by changing culture conditions, it is possible to regulate the MAG content in the diatom *S. marinoi,* especially of MAG-C16 that are specific of diatoms.

In conclusion, our work provides new evidence for the potential benefits of compounds derived from microalgae and their possible applications for human health. This work also highlights, for the first time, the antiproliferative and proapoptotic effect of MAG-ω6 and the diatom-specific MAG-C16, offering new opportunities for the exploitation of microalgal-derived compounds for the prevention and treatment of tumors.

## 4. Materials and Methods 

### 4.1. General

1D and 2D NMR spectra were recorded on a Bruker AVANCE™ III HD-400, equipped with a CryoProbe™ Prodigy or on a Bruker DRX-600 equipped with TXI CryoProbe^TM^ in CDCl_3_ or CDCl_3_/CD_3_OD (δ_H_ values reported refer to CHCl_3_ or CHD_2_OD protons at 7.26 or 3.34 ppm, respectively; δ_C_ values refer to CDCl_3_ or CD_3_OD carbon at 77.0 or 49.0 ppm, respectively). High-resolution mass analyses were performed on a Q-Exactive Hybrid Quadrupole-Orbitrap Mass Spectrometer (Thermo Scientific, Milan, Italy) equipped with a HESI source on-line with a UHPLC Infinity 1290 (Agilent Technologies, Santa Clara, CA, USA). GC-MS analysis was performed on an ion-trap MS instrument in EI mode (70 eV) (Thermo, Polaris Q) connected with a GC system (Thermo, GCQ) by a 5% phenyl/methyl polysiloxane column (30 m × 0.25 mm × 0.25 µm, Agilent, VF-5ms) using helium as the gas carrier. HPLC purifications were carried out on a Shimadzu high-performance liquid chromatography system (Shimadzu, Kyoto, Japan) LC-20ADXR equipped with a Diode Array Detector SPDM-20A and a LUNA C-18(2) column 250 *×* 10 mm, 5 µm, 100 A (Phenomenex, Castel Maggiore, Italy). TLC plates (KieselGel 60 F254) and silica gel powder (Kieselgel 60 0.063–0.200 mm) were from Merck (Darmstadt, Germany). Chemicals were of analytical reagent grade and solvents of HPLC/LC-MS grade (Sigma-Aldrich, Milan, Italy) and were used without any further purification. 

### 4.2. Microalgae Culturing

Diatoms and green algae were grown in Guillard’s f/2 medium [25] (for green algae f/2 without silicates) and dinoflagellates in Keller medium [26] in 10-L polycarbonate bottles with constant bubbling with air filtered through 0.2-µm membrane filters. Cultures were inoculated from healthy exponentially growing cultures at a starting concentration of 2000/3000 cell/mL and kept in a climate chamber at 18 °C, with a 12:12 h light:dark regime, with a light intensity of 100 µmol photons m^−2^ s^−1^ measured at the outside surface of the culture bottle. Cell density was estimated daily using a Sedgewick-Rafter counting chamber (KC, Silkeborg, Denmark) (depth 1 mm) under an optical microscope (Axiovert 200, ZEISS) to obtain growth curves. Cultures were stopped at the end of the stationary phase, since at this stage the production of metabolites is known to increase (e.g., [27]); cells were collected by centrifugation (Biofuge Fresco Heraeus, Cavenago di Brianza (MB), Italy), for 10 min at 1600 g at 4 °C and pellets were immediately frozen in liquid nitrogen and stored at −80 °C until chemical extraction.

### 4.3. Algal Pellet Extraction, Fractionation, and HPLC Isolation of Pure Metabolites

Frozen wet pellets (7.2 g) of *S. marinoi* were extracted by ultrasound three times with methanol (1:5, w:v) at room temperature. The combined organic phases were filtered and the solvent was removed under vacuum by rotavapor, affording a raw extract of 665 mg. Part of the methanol extract (500 mg) was fractionated on a silica gel column by using a stepwise solvent gradient starting from petroleum ether/diethyl ether (EP/EE 90:10) followed by EP/EE 70:30, 40:60, 20:80, and EE 100%. Fraction eluting with 100% EE gave 4.6 mg of a fraction containing monoacylglycerides. Further purification of this fraction was carried out by RP-HPLC on a semipreprative LUNA C18(2) column by using an isocratic elution of 85% MeOH in H_2_O, followed by 100% MeOH column wash, and conditioning at 85% MeOH for 5 min before the successive run. The flow rate was 3 mL/min and peak elution was monitored by a PDA detector at 210 nm. Collected main peaks MAG-16:4 (0.23 mg), MAG-16:3 (0.37 mg), MAG 16:2 (0.55 mg), MAG 16:1 (0.22 mg), and MAG 20:5/MAG18:3 (0.42 mg) were checked by ^1^H-NMR and HRESIMS before further analysis. MAG-16:4 was degraded during sample manipulation.

### 4.4. Cell Cultures and Treatment

The cell lines used to assay the apoptotic properties of the algal extracts were: U-937: histocytic lymphoma cell line grown in RPMI 1640 medium 4.5 g/L glucose (Euroclone, Milan, Italy) supplemented with 10% fetal bovine serum (FBS) (Gibco), 100 U/mL penicillin G and 100 U/mL streptomycin (Lonza, Cologne, Germany), 2 mM L-Glutamine (Lonza, Cologne, Germany ); HCT-116 colon cancer cell line grown in DMEM medium (Sigma-Aldrich) supplemented with 10% FBS, 100 U/mL penicillin G and 100 U/mL streptomycin (Lonza, Cologne, Germany), 2 mM L-Glutamine (Lonza, Cologne, Germany); MePR-2B: primary cell line immortalized from human amniocytes grown in RPMI supplemented with 10% FBS, 100 U/mL penicillin G and 100 U/mL streptomycin (Lonza, Cologne, Germany), and 2 mM L-Glutamine (Lonza, Cologne, Germany) [28,29].

Cells were plated in 24-well culture plates (BD Falcon) at a concentration of 50,000 cells/mL and grown for 24 h at 37 °C and 5% CO_2_ in a fully humidified atmosphere. They were subsequently treated with total algal extract and fractions at increasing concentrations to perform viability and Caspase assays. Commercial MAGs used as standards for the biological assays included: 1-palmitoleoyl-rac-glycerol (MAG-C16:1), 1-Palmitoyl-rac-glycerol (MAG-C16:0), and 1-stearoyl-rac-glycerol (MAG-C18:0) (Sigma-Aldrich, Milan, Italy). SMAR-F2L fraction as well as the purified and commercial MAGs were dissolved in dimethyl sulfoxide (DMSO) for cytotoxicity assays.

### 4.5. Cell Cycle Analysis

Cells were re-suspended in the staining solution containing freshly added RNAse A, propidium iodide (50 mg/mL), sodium citrate (0.1%), and NP40 (0.1%) in PBS solution for 30 min in the dark. Cell cycle distribution was assessed with a FACScalibur flow cytometer (Becton Dickinson), and 10,000 events were analyzed by ModFit version 3 Technology (Verity) and Cell Quest (Becton Dickinson, Milan, Italy) [30,31].

### 4.6. Cell Proliferation Using the Dye Exclusion Test

Cell proliferation assay was performed by trypan blue dye exclusion test. U-937, HCT-116 and MePR-2B cells (1 × 10^4^ cells/mL) were plated into 96-well in triplicate and administered vehicle (DMSO), purified, synthetic, and commercial MAGs, as well as SMAR-F2L fraction at the concentration of 25 µg/mL. Cells were treated for 24 or 48 h, and afterwards were diluted in the ratio 1:1 in Trypan blue (Sigma-Aldrich) and counted with an optical microscope in order to discriminate dead cells (blue) from living cells, which do not stain.

### 4.7. Caspase 3/7 Assay

Caspase activity was detected within living cells using the FAM FLICA Caspase 3&7 kit (Biocompare) with cell-permeable fluorescent substrates following the manufacturer’s instructions. The fluorescent substrates for caspase-3/7 were FAM-DEVD-FMK. Cells were washed twice in cold PBS 1X and incubated for 1 h in ice with the corresponding substrate, as recommended by the suppliers. Cells were analyzed using BD CellQuest Pro software version 5.2 (San Jose, CA 95131-1807, USA) applied to a FACScalibur (BD Biosciences, Milan, Italy). Experiments were performed in triplicate and values are expressed as mean +/− SD.

### 4.8. MTT Assay

The effect of MAGs on cell viability was determined by the MTT (3-(4, 5-dimethyl thiazol-2yl)-2, 5-diphenyl tetrazolium bromide) assay. Cells were seeded in a 96-well flat-bottom plate at a density of 5 × 10^3^ cells/well for 24 h at 37 °C in a CO_2_ incubator. After 24 h of incubation, the culture medium was replaced with a fresh medium. Cells were then treated with increasing concentrations of commercial and purified MAGs for 24 and 48 h at 37 °C in a CO_2_ incubator. Subsequently, 10 μL of MTT working solution (5 mg/mL in phosphate buffer solution) were added to each well and the plate was incubated for 4 h at 37 °C in a CO_2_ incubator.

The medium was then aspirated, and the formed formazan crystals were solubilized by adding 50 μL of DMSO. Absorbance intensity was measured using an infinite M200 plate reader (TECAN) at O.D. = 570 nm. Experiments were performed in triplicate and values are expressed as mean +/− SD.

### 4.9. Statistical Analysis

ANOVA (α = 0.01) with Tukey’s post-hoc tests were used to determine significant differences in the percentage of cells in sub-G1 treatments between each extract or compound with respect to relative controls (Figure 1 and Figure 2; Supplementary Figure S1). The same tests were used to determine statistical differences between treated and control cells for cell viability experiments (Figure 6; Supplementary Figures S10–S12) as well as for caspase activity (Figure 7). *p*-value < 0.01 were considered significant.

All statistical analyses were performed using GraphPad Prism 5.0 (GraphPad Software, Inc., San Diego, CA, USA).

### 4.10. LC-MS Analysis of Methanol Extract and Bioactive Fraction from Skeletonema marinoi

Both methanol extract (250 µg/mL in MeOH) and bioactive fraction (100 µg/mL in MeOH) were analyzed by UHPLC-MS/MS (10-µL sample injection volume) on a Q-Exactive platform (Thermo Scientific, San Jose, CA, USA) following the method reported in Cutignano et al. [32]. Briefly, the column used for chromatographic separation was a Kinetex Biphenyl 2.6 µm, 150 × 2.1 mm column (Phenomenex, Castel Maggiore, Bologna, Italy) kept at 28 °C. Eluent A: water and eluent B: MeOH. The elution program consisted of a gradient from 40% to 80% B in 2 min, then to 100% B in 13 min, holding at 100% B for 7 min. The flow rate was 0.3 mL/min. Full MS scans were acquired in positive polarity over the range *m/z* 200–1200. A targeted MS/MS method was used for ion fragmentation. A stepped normalized energy of 25–28–35% was applied. 

### 4.11. GC-MS Analysis of FAME 

A small aliquot of the extract (5 mg) was subjected to methanolysis by Na_2_CO_3_ in MeOH (1 mL) at 45 °C overnight under stirring. The reaction mixture was successively diluted with milliQ water, neutralized with HCl 1M, and extracted with EE for three times (3 × 2 mL). Combined extracts were dried under a nitrogen stream, resuspended in MeOH at a final concentration of 1 mg/mL, and analyzed by GC-MS (2-µL sample injection volume) using the following temperature gradient: Initial 160 °C holding for 3 min; then 5 °C/min up to 260 °C followed by 30 °C/min up to 310 °C, holding for 3 min at 310 °C; split flow 10 mL/min; full scan *m/z* 50–450.

### 4.12. Synthesis and Characterization of 1-O-Arachidonoyl Glycerol (1-MAG-ARA)

**Scheme 1:**
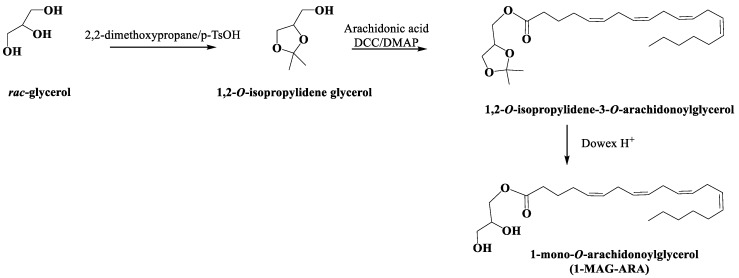
Synthetic route for the preparation of 1-mono-*O*-arachidonoylglycerol (1-MAG-ARA).

All the synthetic intermediates were characterized by 1D-and 2D-NMR analysis. The synthetic route to obtain 1-AG is reported in Scheme 1.

1,2-*O*-isopropylidene glycerol: Glycerol (1.5 g, 0.017 mol) was dissolved in anhydrous *N,N*-dimethylformamide (3 mL); 2,2-dimethoxypropane (3 mL) and *p*-toluensulfonic acid (225 mg) were added; after stirring overnight at room temperature, the mixture was portioned between water and dichloromethane; the organic phase was purified by silica gel chromatography using a gradient of petroleum ether/diethyl ether to give 1,2-*O*-isopropylidene glycerol (1.5 g, 0.011 mol, 68%). ^1^H-NMR (CDCl_3_): *δ* 4.24 (1H, m, H-2), 4.09 (1H, dd, *J* = 6.7, 8.5 Hz, H-1a), 3.82 (1H, dd, *J* = 6.4, 8.5 Hz, H-1b), 3.65 (2H, m, H_2_-1), 1.46 (3H, s, CH_3_), 1.40 (3H, s, CH_3_).

1,2-*O*-isopropylidene-3-*O*-arachidonoylglycerol: 1,2-*O*-isopropylidene glycerol (0.325 g, 0.00077 mol) was dissolved in anhydrous dichloromethane (5 mL); arachidonic acid (0.279 g, 0.00092 mol), *N,N*-dicyclohexylcarbodiimide (0.188 g, 0.00092 mol), and *N,N*-dimethylaminopyridine (0.006 g, 0.00005 mol) were added under argon and the reaction mixture was stirred overnight at room temperature; after evaporation under reduced pressure, the mixture was purified by silica gel chromatography using a gradient of petroleum ether/diethyl ether to give 1,2-*O*-isopropylidene-3-*O*-arachidonoylglycerol (0.301 g, 0.00072 mol, 93%). 

1-mono-*O*-arachidonoylglycerol (1-MAG-ARA): 1,2-*O*-isopropylidene-3-*O*-arachidonoylglycerol (0.301 g, 0.00072 mol) was dissolved in methanol/chloroform/water mixture (3/3/1) (7 mL) and Dowex H^+^ resin (8 g) was added; the mixture was stirred at room temperature for 3 h. After filtration, the filtrate was purified by silica gel chromatography using a gradient of petroleum ether/diethyl ether to give the mono-*O*-arachidonoylglycerol (1-MAG-ARA) (0.15 g, 0.00040 mol, 56%) that was further purified on reversed phase HPLC (Synergi Fusion Rp column Phenomenex) with a gradient of elution of methanol and water; the gradient started from 80% to 100% of methanol in 10 min; IR (film) ν_max_ 3480, 3200, 1748, 1650, 1420 cm^−1^; ^1^H-NMR (600 MHz; CD_3_OD/CDCl_3_ 1/1): δ 5.37-5.31 (8H, vinyl protons), 4.13 (1H, dd, *J* = 4.4, 11.2 Hz), 4.07 (1H, dd, *J* = 6.0, 11.2 Hz), 3.83 (1H, m), 3.58 (1H, dd, *J* = 4.8, 11.2 Hz), 3.53 (1H, dd, *J* = 5.9, 11.2 Hz), 2.86–2.71 (6H, m), 2.35 (2H, t, *J* = 7.7 Hz, α-methylene of arachidonoyl portion), 2.16–2.01 (4H, allylic protons), 1.68 (2H, m, β-methylene of arachidonoyl portion), 1.36–1.24 (aliphatic protons), 0.86 (3H, t, *J* = 6.68 Hz). ^13^C-NMR (100 MHz, CD_3_OD/CDCl_3_ 1/1): δ 175.2 (C, acyl ester), 129.6–129.4 (8CH, vinylic carbons), 70.2 (CH, glycerol carbon 2), 67.2 (CH_2_, glycerol carbon 1), 64.5 (CH_3_, glycerol carbon 3), 34.4 (CH_2_, α-methylene of arachidonoyl portion), 28.5, 27.4 (2CH_2_, allylic carbons), 29.8–27.8 (3CH_2_, alyphatic carbons), 26.6 (3CH_2_, bisallylic carbons), 26.5 (CH_2_, β-methylene of arachidonoyl portion); HR-ESIMS *m/z* 401.2664 [M + Na]^+^.

## Supplementary Materials

The following data are available online at https://www.mdpi.com/1660-3397/17/11/625/s1, Figure S1: Percentage of cells in the sub-G1 phase after 24 and 72 h of treatment with silica fraction SMAR-F2L at increasing concentrations of 10, 30, and 90 µg/mL in U-937, HCT-116, and MePR-2B cell lines; Figure S2: ^1^H, ^1^H COSY-NMR spectrum (400 MHz, CDCl_3_) of fraction SMAR-F2L; Figure S3: ^1^H, ^1^H TOCSY-NMR spectrum (400 MHz, CDCl_3_) of fraction SMAR-F2L; Figure S4: Edited-HSQC NMR spectrum (400 MHz, CDCl_3_) of fraction SMAR-F2L; Figure S5: HMBC-NMR spectrum (400 MHz, CDCl_3_) of fraction SMAR-F2L; Figure S6: ^1^H-NMR spectrum (600 MHz, CDCl_3_) of MAG-C16:4; Figure S7: ^1^H-NMR spectrum (600 MHz, CDCl_3_) of MAG-C16:3; Figure S8: ^1^H-NMR spectrum (600 MHz, CDCl_3_) of MAG-C16:2; Figure S9: ^1^H-NMR spectrum (600 MHz, CDCl_3_) of MAG mixture EPA/C18:3; Figure S10: Cell viability assay (MTT) on U-937 (A) and HCT-116 (B) cell lines after 24 h of treatment with purified and commercial MAGs at increasing concentrations of 5, 10, 25, 35, and 50µg/mL; Figure S11: Cell viability assay (MTT) on MePR-2B cells after 24 and 48 h of treatment with purified and commercial MAGs at increasing concentrations of 5, 10, 25, 35, and 50 µg/mL; Table S1. List of species selected from the culture collection of the Stazione Zoologica with the relative identification code; Table S2: Fatty acid composition of *Skeletonema marinoi* lipids by GC-MS analysis of FAME derivatives. Table S3: Statistical results for two-way ANOVA, Tukey post-test (α = 0.051) performed on proliferation assay for U-937, HCT-116, and MePR-2B cells treated with 25 µg/mL of purified and commercial MAGs.
