# Supplementary material for: Monoacylglycerides from the Diatom Skeletonema marinoi Induce Selective Cell Death in Cancer Cells
Source: Mar Drugs. 2019 Nov 1;17(11):625. doi: 10.3390/md17110625 (PMC6891494; doi:10.3390/md17110625)
Supplement: Supplementary file 1 [file marinedrugs-17-00625-s001.pdf]

Monoacylglycerides from the diatom *Skeletonema marinoi* induce selective cell death in cancer cells

Marco Miceli [1,§], Adele Cutignano [2,§], Mariarosaria Conte [3,§], Raffaella Ummarino [4,6], Alessandra Romanelli [5], Menotti Ruvo [4], Marilisa Leone [4], Flavia Anna Mercurio [4], Nunzianna Doti [4], Emiliano Manzo [2], Giovanna Romano [6,*], Lucia Altucci [3,*] and Adrianna Ianora [6].

Supplementary Information

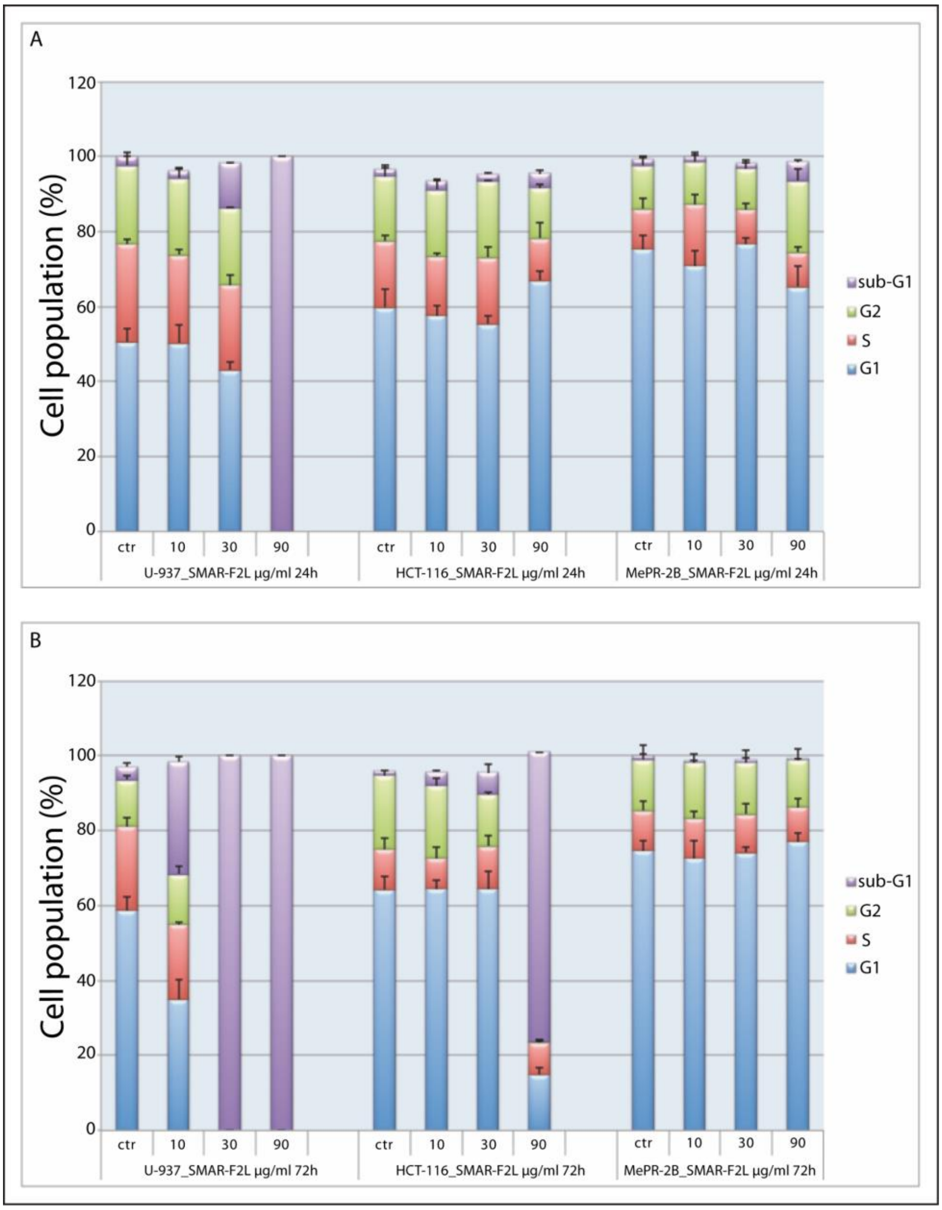

Figure S1. Cell cycle distribution in U-937, HCT-116 and MePR-2B cells after treatment for 24 (A) and 72 hours (B) with fraction SMAR-F2L at 10, 30 and 90µg/ml with the vehicle DMSO (control). All bars represent means ± SD with n = 3.

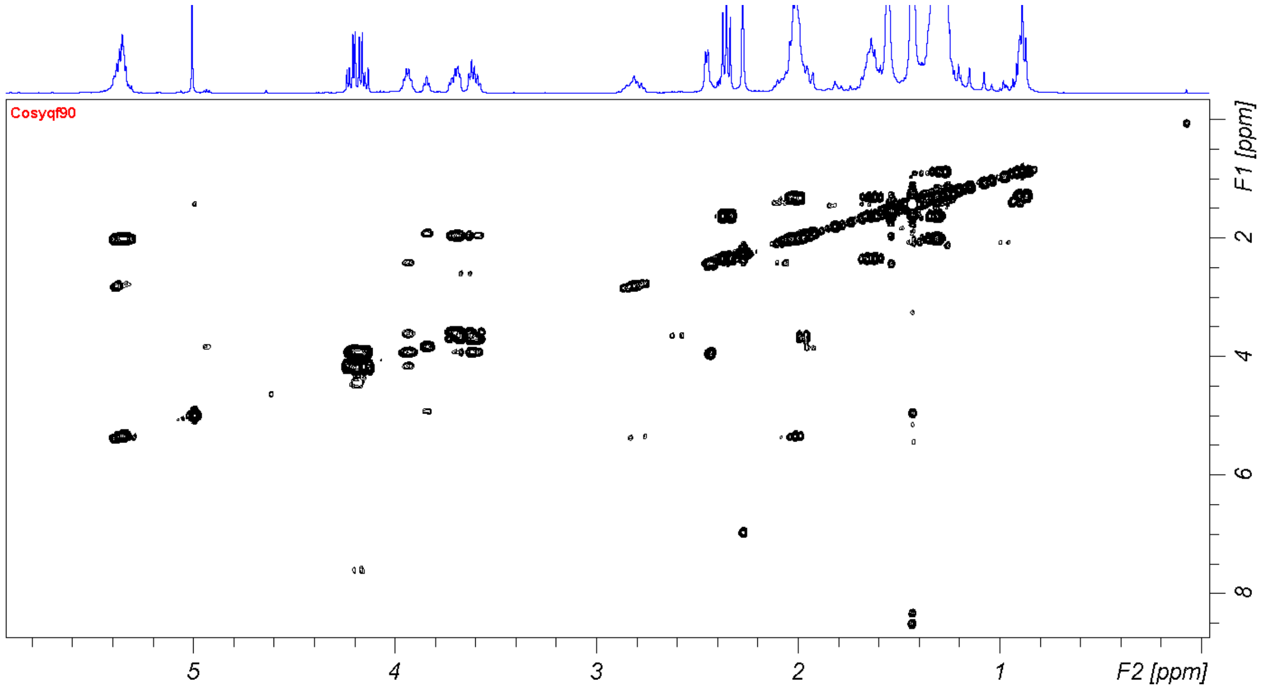

Figure S2. ^{1}H, ^{1}H COSY-NMR spectrum (400MHz, $CDCl_3$) of fraction SMAR-F2L.

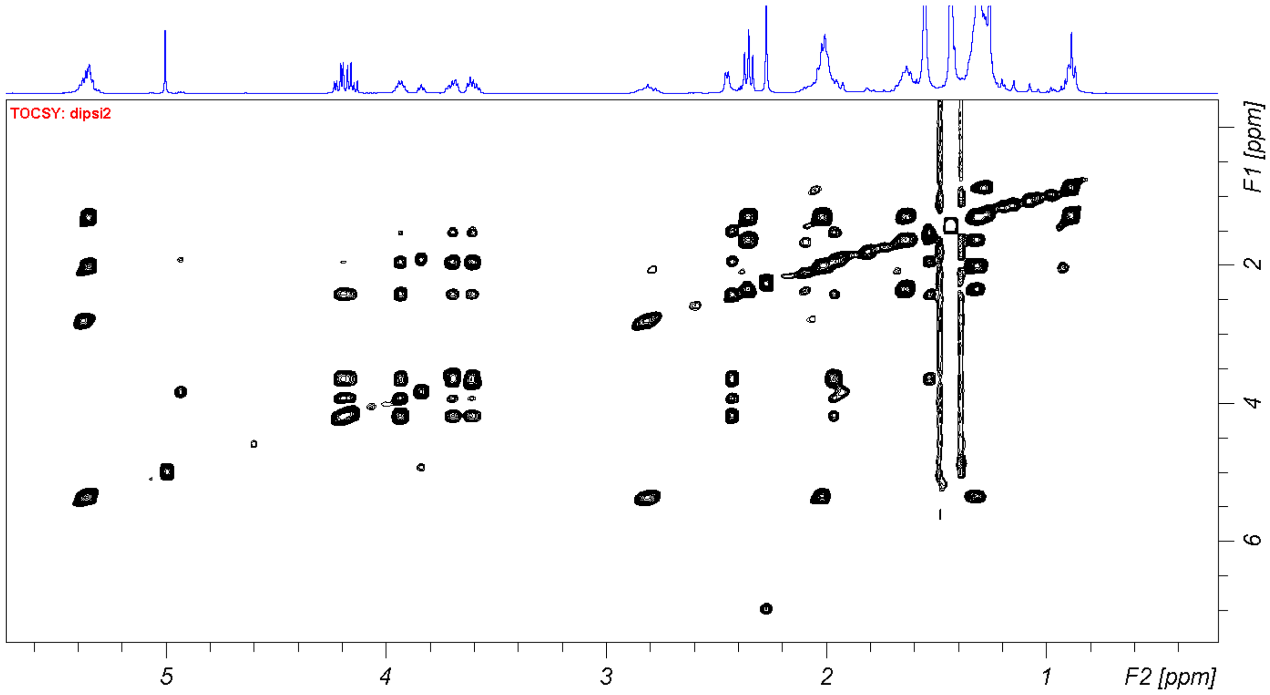

Figure S3. ^{1}H, ^{1}H TOCSY-NMR spectrum (400MHz, $CDCl_3$) of fraction SMAR-F2L.

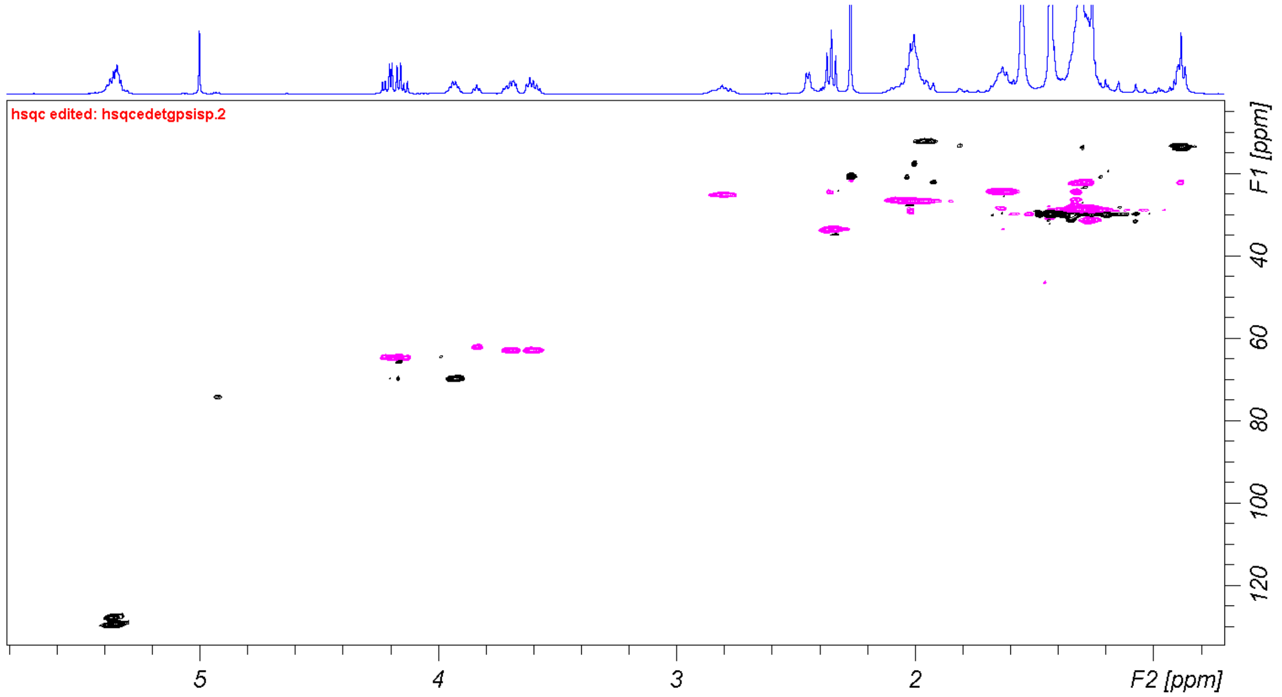

Figure S4. Edited-HSQC NMR spectrum (400MHz, $CDCl_3$) of fraction SMAR-F2L.

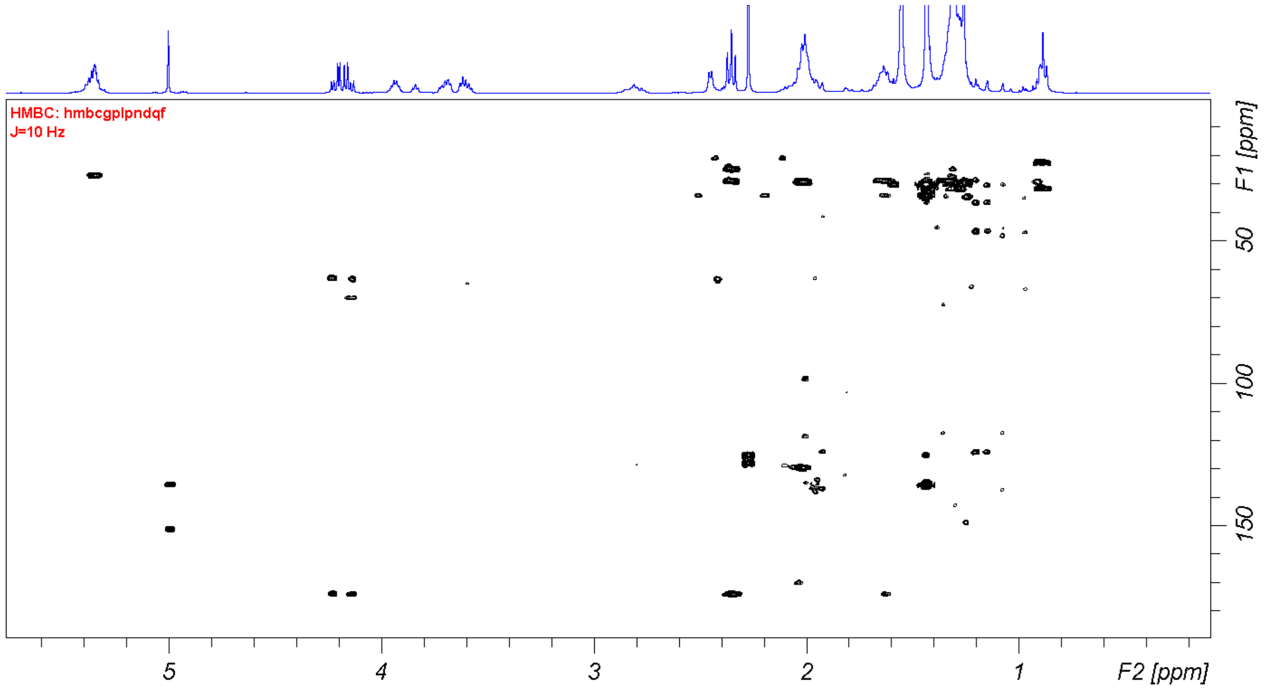

Figure S5. HMBC-NMR spectrum (400MHz, $CDCl_3$) of fraction SMAR-F2L.

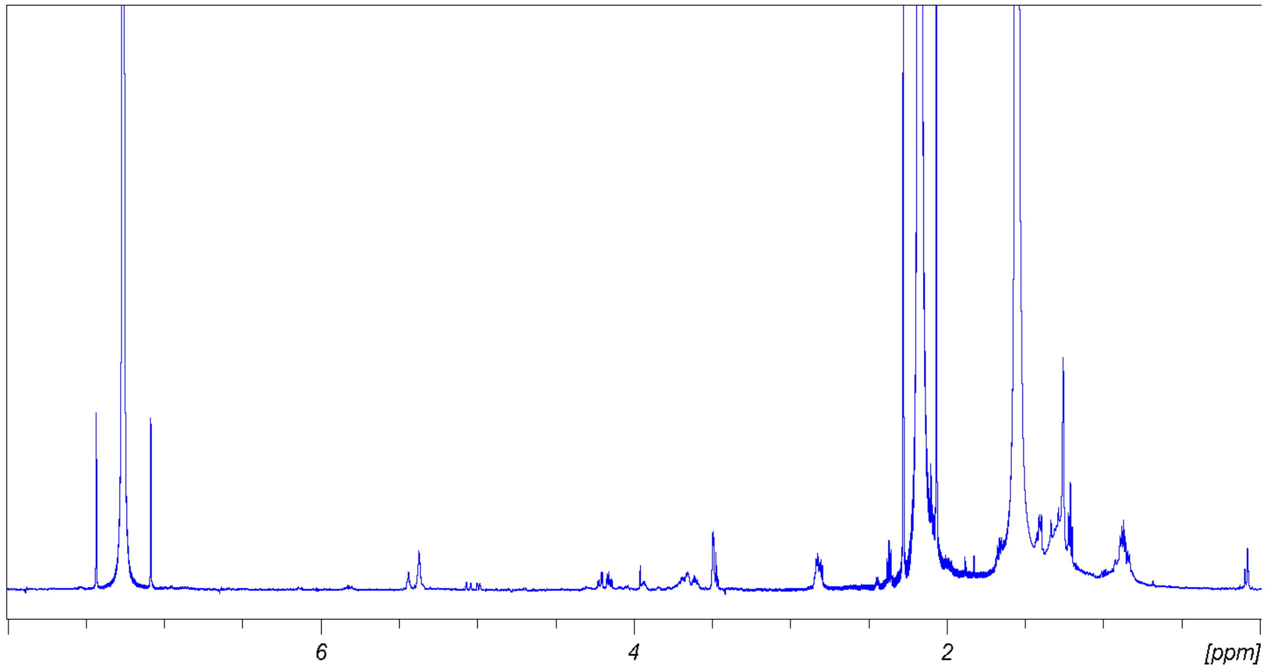

Figure S6. ^{1}H-NMR spectrum (600MHz, $CDCl_3$) of MAG-C16:4.

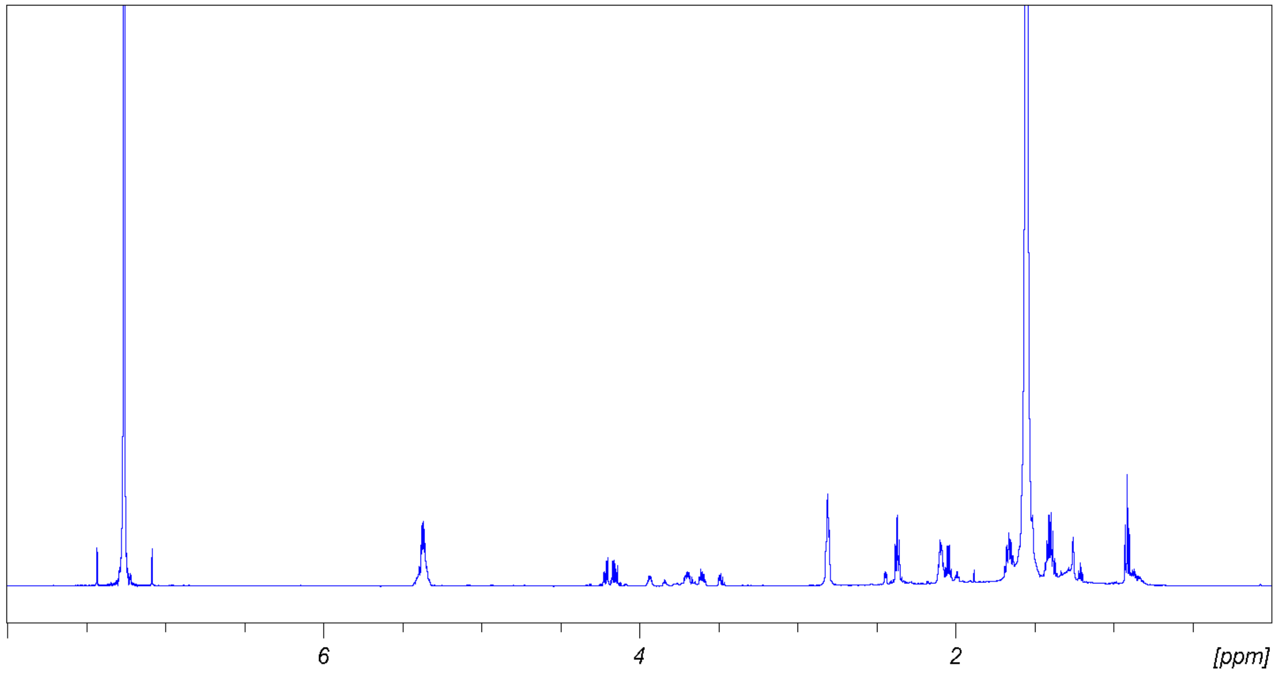

Figure S7. ^{1}H-NMR spectrum (600MHz, $CDCl_3$) of MAG-C16:3

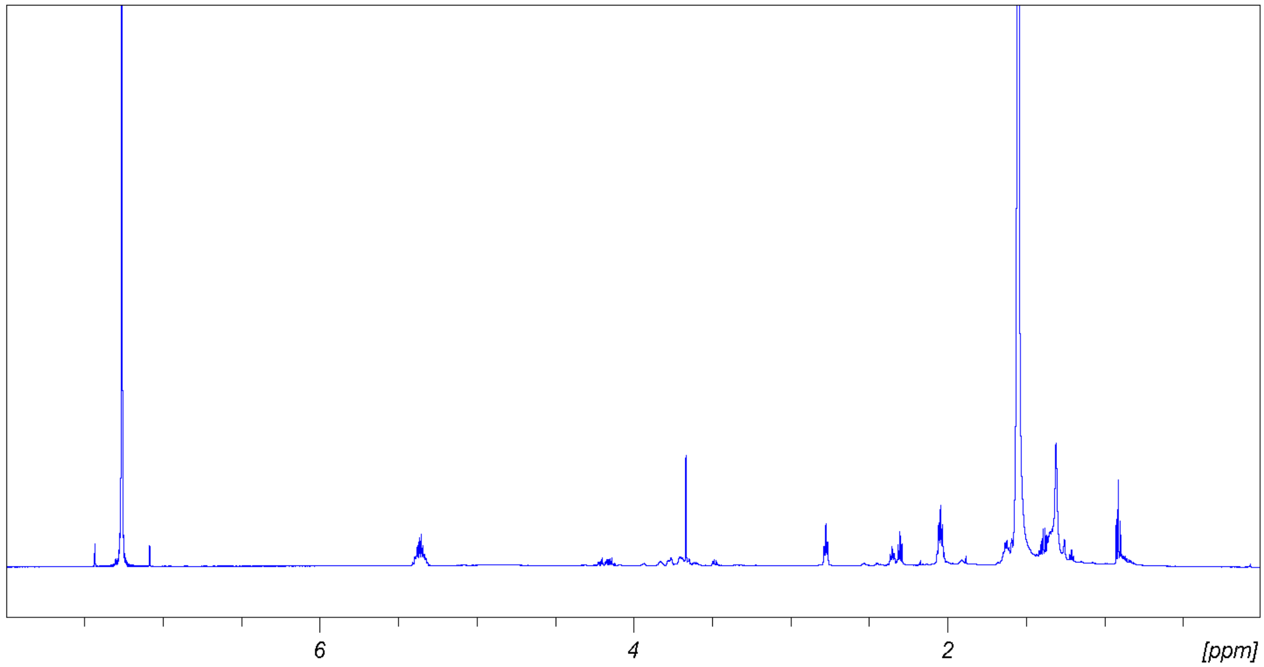

Figure S8. ^{1}H-NMR spectrum (600MHz, $CDCl_3$) of MAG-C16:2

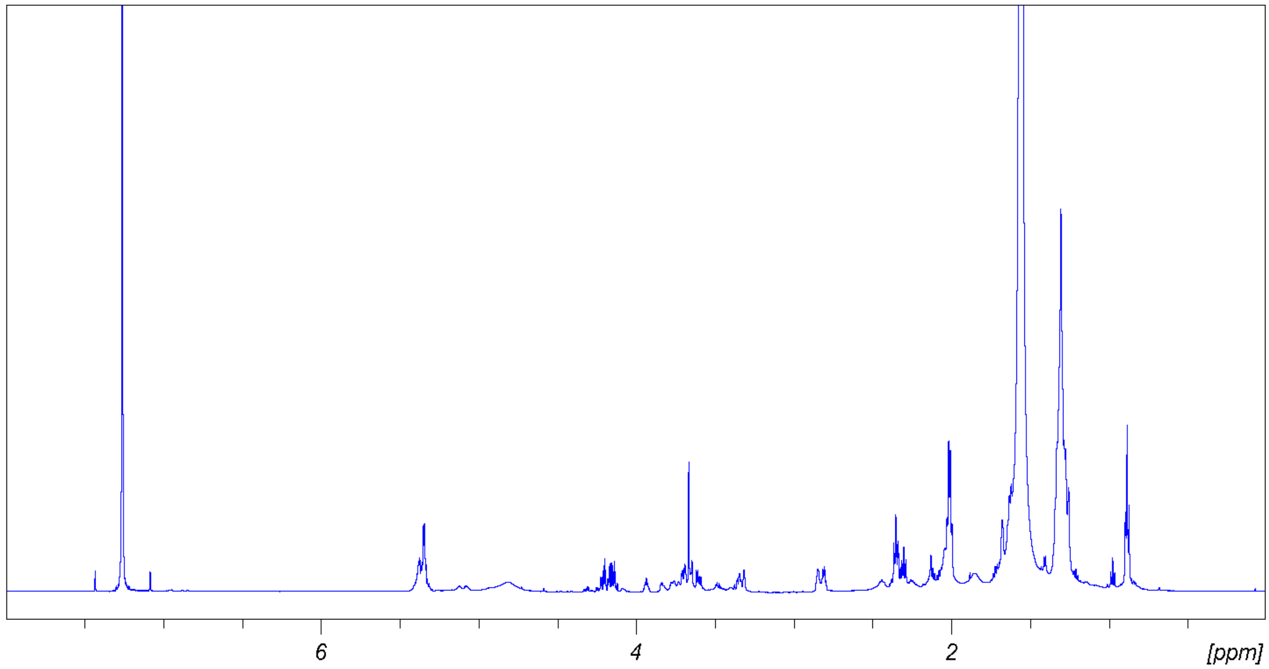

Figure S9. ^{1}H-NMR spectrum (600MHz, $CDCl_3$) of MAG mixture EPA/C18:3.

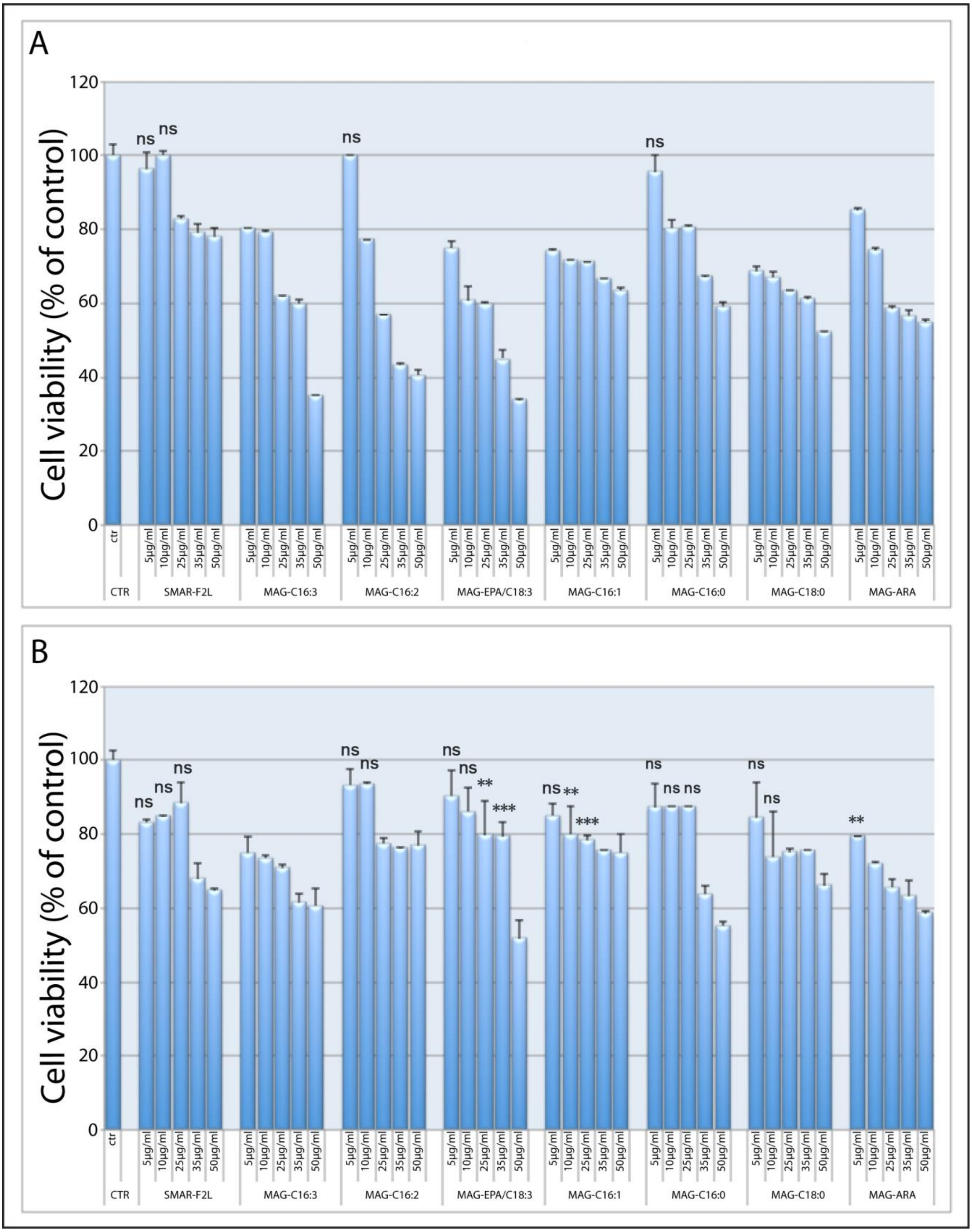

Figure S10. Cell viability assay (MTT) on U-937 (A) and HCT-116 (B) cell lines after 24 hours of treatment with purified and commercial MAGs at increasing concentrations of 5, 10, 25, 35 and 50µg/ml. All bars represent means ± SD with n = 3.

Only differences non-significant with respect to the control (ns) and difference with p-value<0.05 (*), p<0,01 (**) and p<0,001 (***) are indicated. Extremely significant differences (p<0,0001), which are the majority, are not indicated to avoid overcrowding of symbols (ANOVA, with Tukey's test; p<0.01 was considered significant).

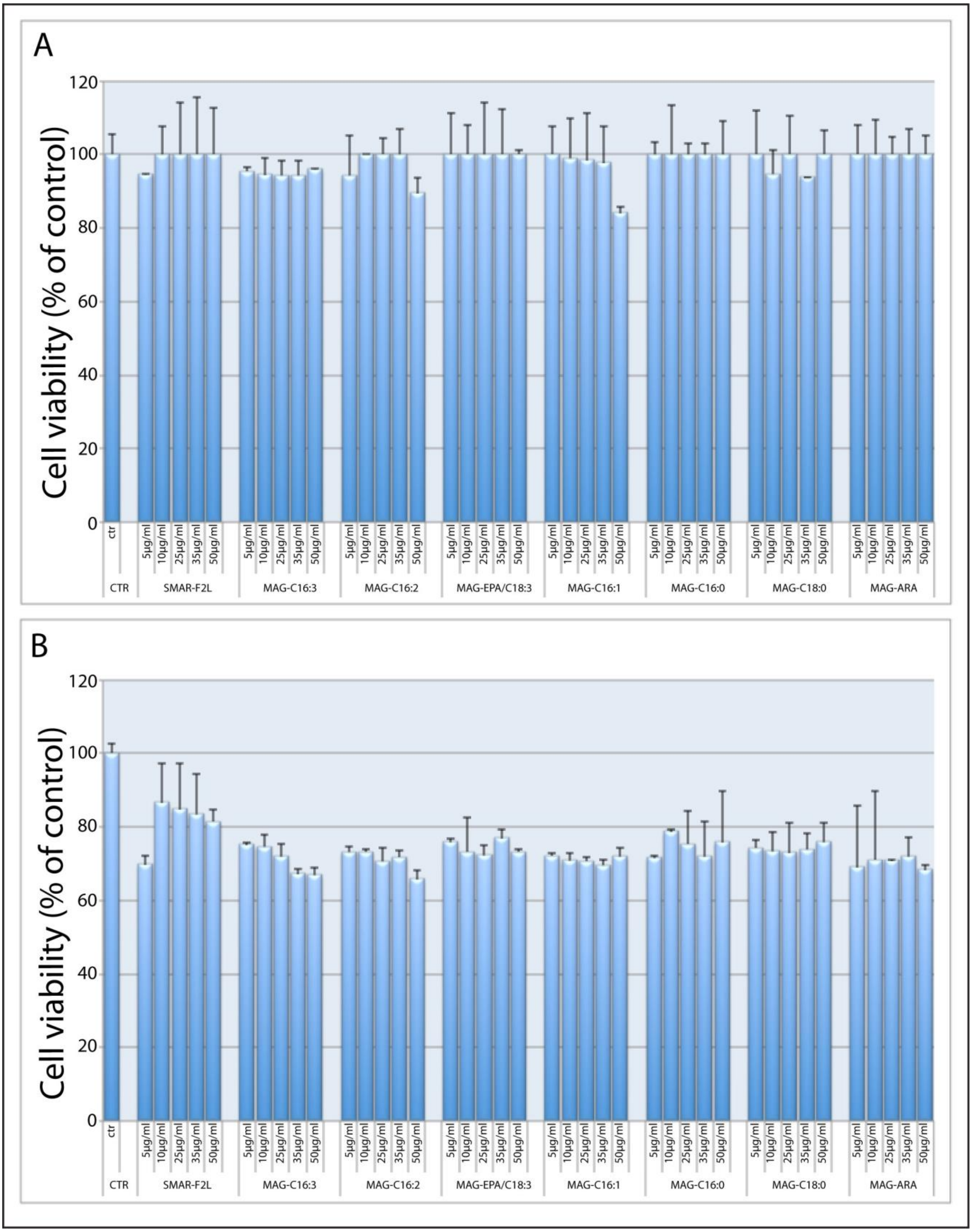

Figure S11. Cell viability assay (MTT) on MePR-2B cells after 24 (A) and 48 hours (B) of treatment with purified and commercial MAGs at increasing concentrations of 5, 10, 25, 35 and 50μg/ml. All bars represent means ± SD with n = 3. No significant difference was observed (ANOVA, with Tukey's test; p<0.01 was considered significant)

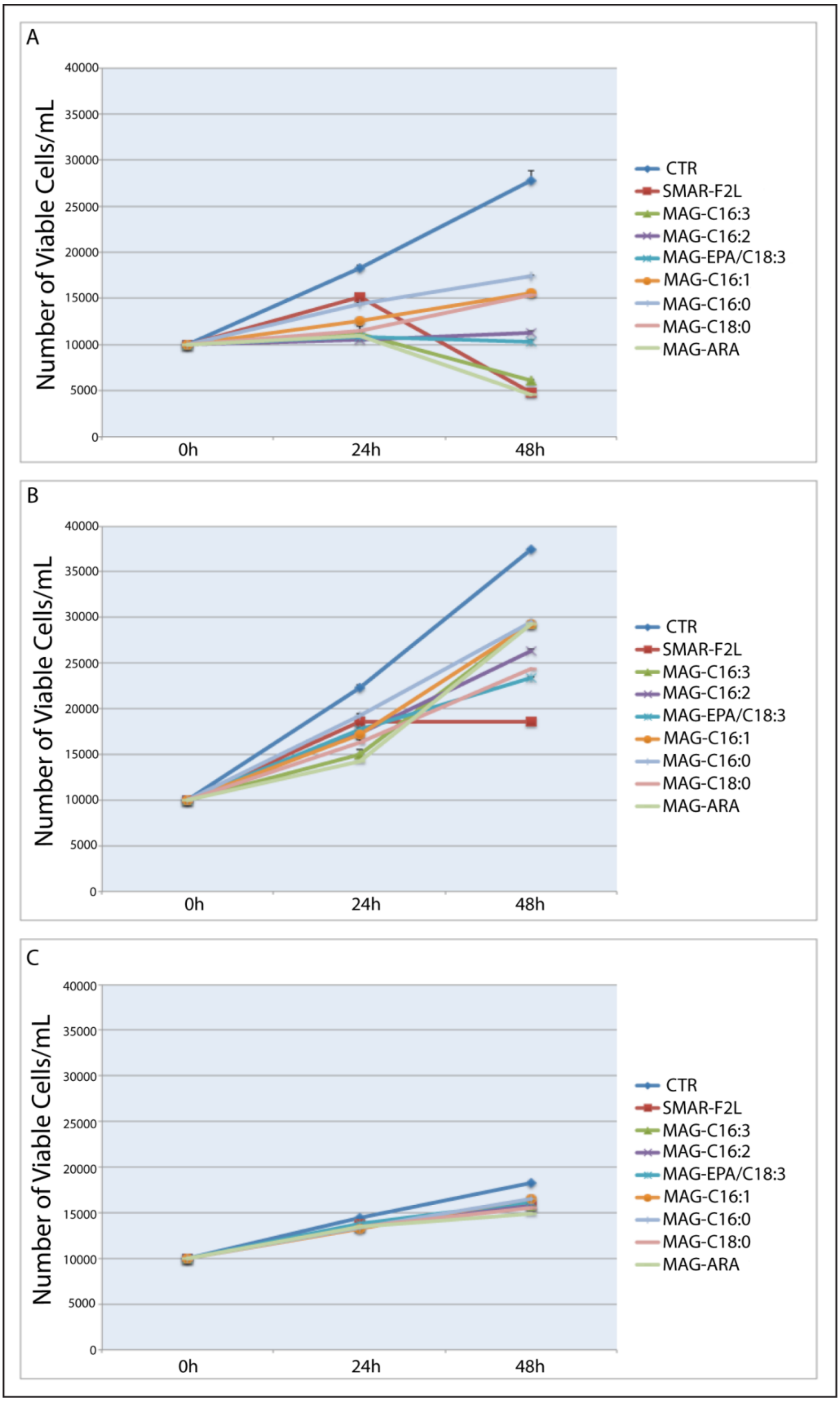

Figure S12. Cell proliferation assay on U-937 (A), HCT-116 (B) and MePR-2B (C) cells after 24 and 48 hours of treatment with purified and commercial MAGs at the concentration of 25μg/ml. All points represent means ± SD with n = 3. Statistical results of Two-Way ANOVA with Tukey's post-test are reported in supplementary table S2.

Table S1. List of species selected from the culture collection of the Stazione Zoologica with relative identification code.

Code	Species
FE7	*Skeletonema marinoi*
FE102	*Amphidinium carterae*
FE80	*Thalassiosira rotula*
FE205	*Tetraselmis suecica*
FE107	*Alexandrium tamarense*
FE1320	*Dunaliella salina*
FE21	*Chaetoceros affinis*
FE32	*Skeletonema japonicum*

Table S2. Fatty acid composition of *Skeletonema marinoi* lipids by GC-MS analysis of FAME derivatives.

**FAME**	**Apex RT**	**%Area**		
**C14:0**	**8,96**	**4,79**		
**C16:4w1**	**13,06**	**2,16**		
**C16:3w4**	**13,22**	**16,23**		
**C16:1+C16:2**	**13,59**	**34,06**		
**C16:0**	**14,17**	**5,1**		
**C18:3**	**18,39**	**0,05**		
**C18:4w3**	**18,53**	**6,81**		
**C18:2w6**	**18,85**	**0,76**		
**C18:1w9**	**19,03**	**0,52**		
**C18:1w7**	**19,21**	**0,4**		
**C18:0**	**19,82**	**1,59**		
**ARA**	**23,35**	**0,06**		

Table S3. Statistical results for Two-Way ANOVA, Tukey post-test (α = 0.01) performed on proliferation assay for U-937, HCT-116 and MePR-2B cells treated with 25 μg/ml of purified and commercial MAGs (Supplementary Figure S12). P-values<0.01 were considered significant.

Tukey's multiple comparisons test	Mean Diff	95,00% CI of diff	Summary	Adjusted p-Value
**U-937 24 h**				
ctr dmso vs. SMAR-F2L	1475	123,6 to 2826	*	0,0229
ctr dmso vs. MAG-C16:3	5070	3719 to 6421	****	<0,0001
ctr dmso vs. MAG-C16:2	5790	4439 to 7141	****	<0,0001
ctr dmso vs. MAG-EPA/C18:3	5495	4144 to 6846	****	<0,0001
ctr dmso vs. MAG-C16:1	3715	2364 to 5066	****	<0,0001
ctr dmso vs. MAG-C16:0	1905	553,6 to 3256	**	0,0011
ctr dmso vs. MAG-C18:0	4775	3424 to 6126	****	<0,0001
ctr dmso vs. MAG-ARA	5345	3994 to 6696	****	<0,0001
**U-937 48 h**				
ctr dmso vs. SMAR-F2L	19190	17839 to 20541	****	<0,0001
ctr dmso vs. MAG-C16:3	17888	16536 to 19239	****	<0,0001
ctr dmso vs. MAG-C16:2	12745	11394 to 14096	****	<0,0001
ctr dmso vs. MAG-EPA/C18:3	13645	12294 to 14996	****	<0,0001
ctr dmso vs. MAG-C16:1	8383	7032 to 9734	****	<0,0001
ctr dmso vs. MAG-C16:0	6598	5247 to 7949	****	<0,0001
ctr dmso vs. MAG-C18:0	8583	7231 to 9934	****	<0,0001
ctr dmso vs. MAG-ARA	19459	18108 to 20810	****	<0,0001
**HCT-116 24 h**				
ctr dmso vs. SMAR-F2L	1700	1047 to 2353	****	<0,0001
ctr dmso vs. MAG-C16:3	5300	4647 to 5952	****	<0,0001
ctr dmso vs. MAG-C16:2	2969	2316 to 3622	****	<0,0001
ctr dmso vs. MAG-EPA/C18:3	2539	1886 to 3191	****	<0,0001
ctr dmso vs. MAG-C16:1	3050	2397 to 3702	****	<0,0001
ctr dmso vs. MAG-C16:0	1007	354,0 to 1660	***	0,0004
ctr dmso vs. MAG-C18:0	3997	3344 to 4650	****	<0,0001
ctr dmso vs. MAG-ARA	6015	5362 to 6668	****	<0,0001
**HCT-116 48 h**				
ctr dmso vs. SMAR-F2L	17960	17307 to 18613	****	<0,0001
ctr dmso vs. MAG-C16:3	7260	6607 to 7913	****	<0,0001
ctr dmso vs. MAG-C16:2	10210	9557 to 10863	****	<0,0001
ctr dmso vs. MAG-EPA/C18:3	13210	12557 to 13863	****	<0,0001
ctr dmso vs. MAG-C16:1	7270	6617 to 7923	****	<0,0001
ctr dmso vs. MAG-C16:0	7078	6425 to 7730	****	<0,0001
ctr dmso vs. MAG-C18:0	12193	11540 to 12845	****	<0,0001
ctr dmso vs. MAG-ARA	7255	6602 to 7908	****	<0,0001
**MePR-2B 24 h**				
ctr dmso vs. SMAR-F2L	-245	-1145 to 654,9	ns	0,9917
ctr dmso vs. MAG-C16:3	35	-864,9 to 934,9	ns	>0,9999
ctr dmso vs. MAG-C16:2	91,5	-808,4 to 991,4	ns	>0,9999
ctr dmso vs. MAG-EPA/C18:3	-238	-1138 to 661,9	ns	0,9932
ctr dmso vs. MAG-C16:1	236,5	-663,4 to 1136	ns	0,9934
ctr dmso vs. MAG-C16:0	198	-701,9 to 1098	ns	0,9981

ctr dmso vs. MAG-C18:0	77,5	-822,4 to 977,4	ns	>0,9999
ctr dmso vs. MAG-ARA	98	-801,9 to 997,9	ns	>0,9999
**MePR-2B 48 h**				
ctr dmso vs. SMAR-F2L	1649	748,6 to 2548	****	
ctr dmso vs. MAG-C16:3	1715	815,1 to 2615	****	<0,0001
ctr dmso vs. MAG-C16:2	1694	794,1 to 2594	****	<0,0001
ctr dmso vs. MAG-EPA/C18:3	1233	332,6 to 2132	**	<0,0001
ctr dmso vs. MAG-C16:1	983	83,06 to 1883	*	0,0019
ctr dmso vs. MAG-C16:0	950	50,06 to 1850	*	0,0235
ctr dmso vs. MAG-C18:0	1904	1004 to 2804	****	0,0319
ctr dmso vs. MAG-ARA	2605	1705 to 3505	****	<0,0001
